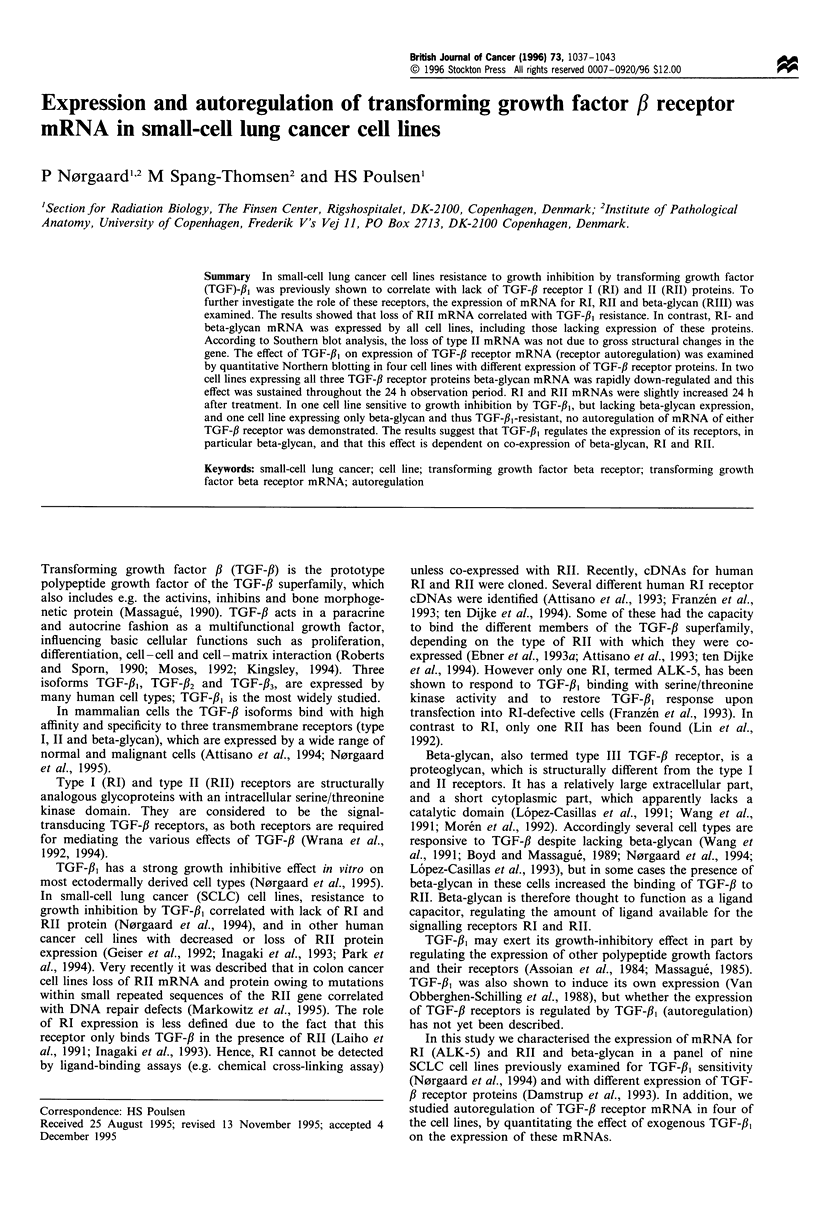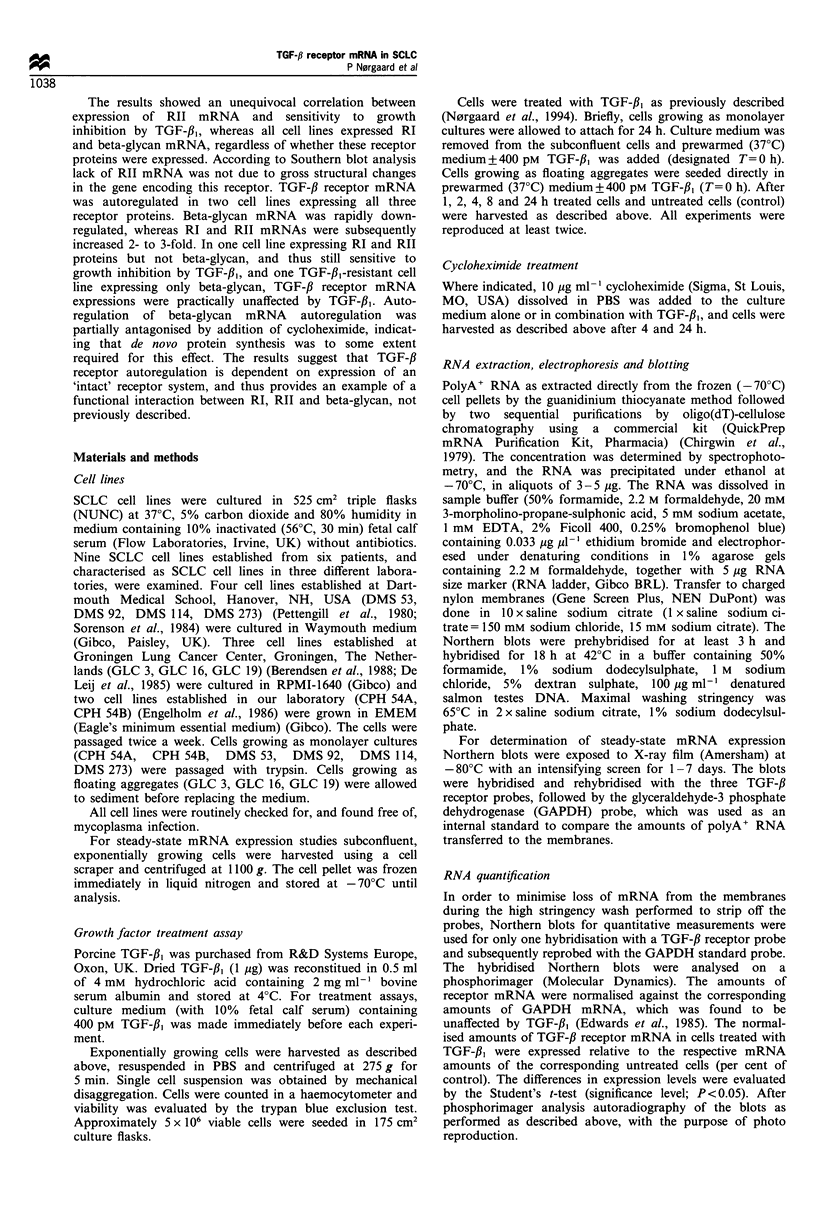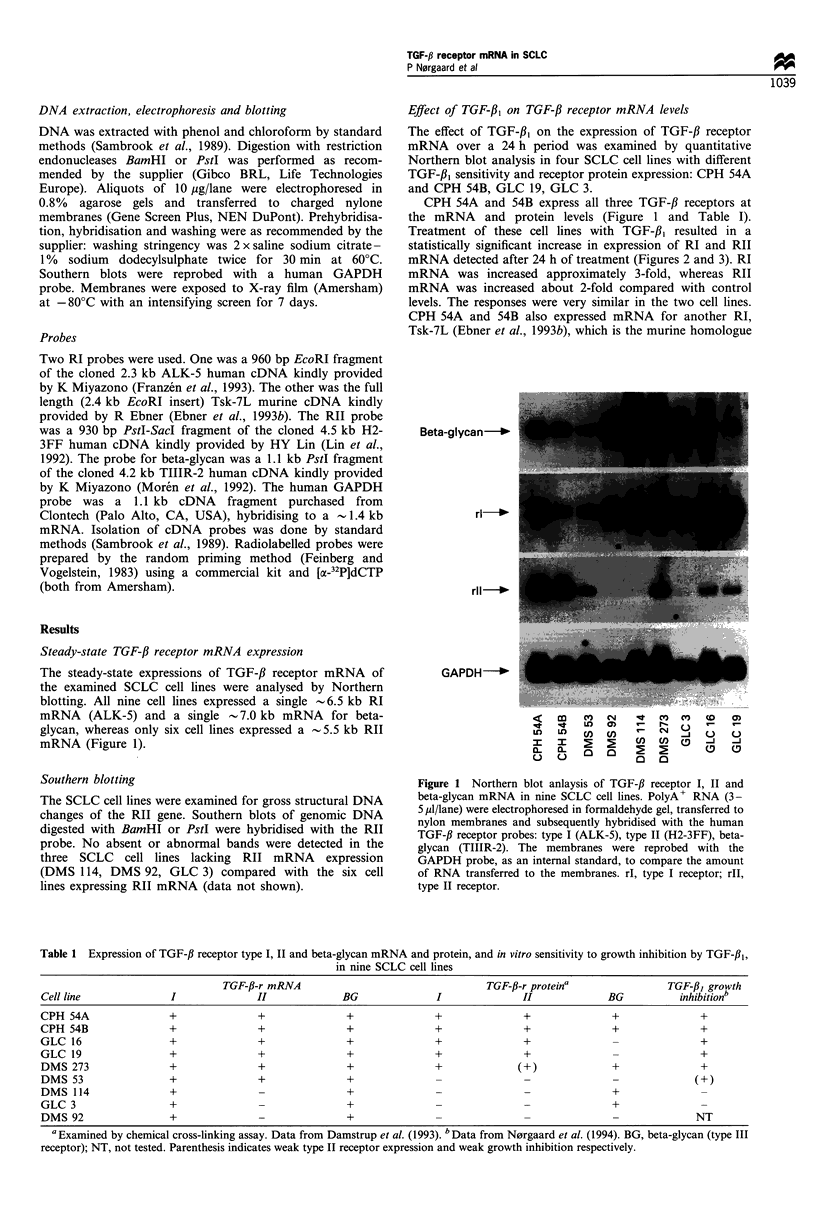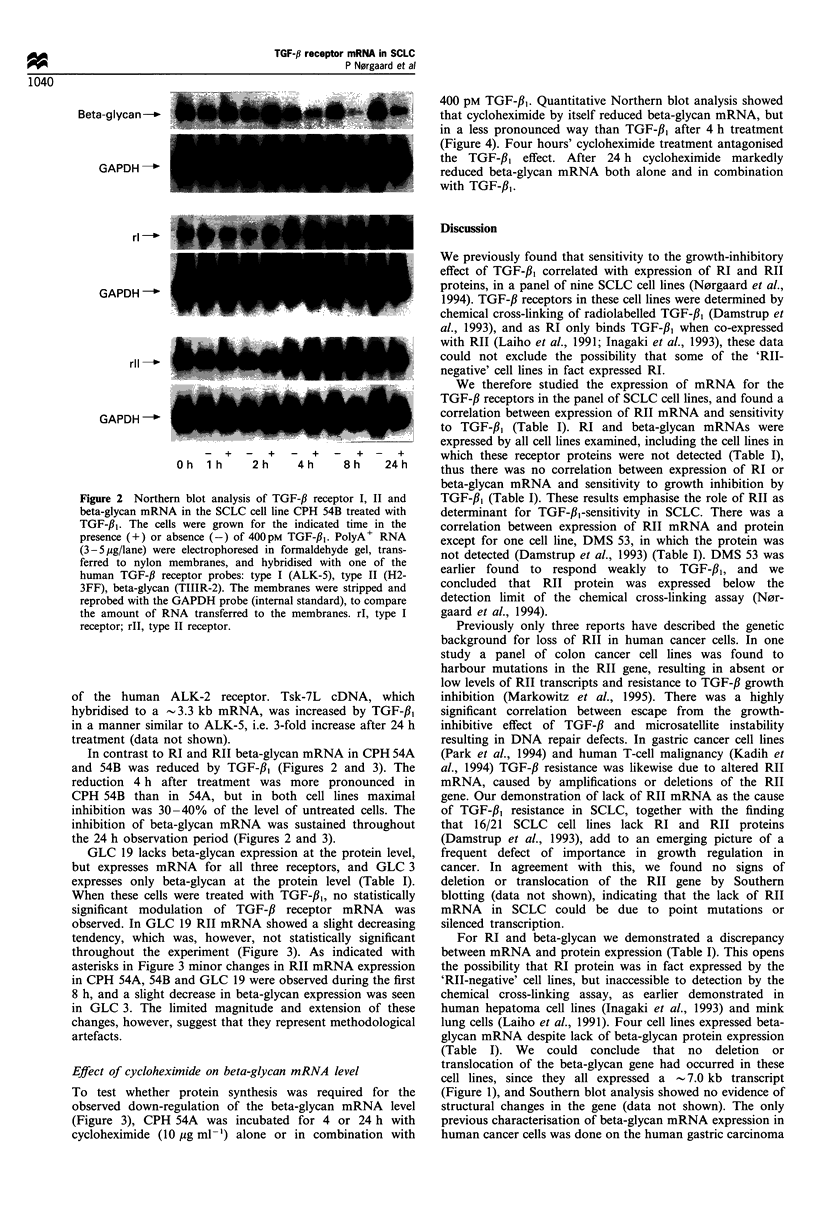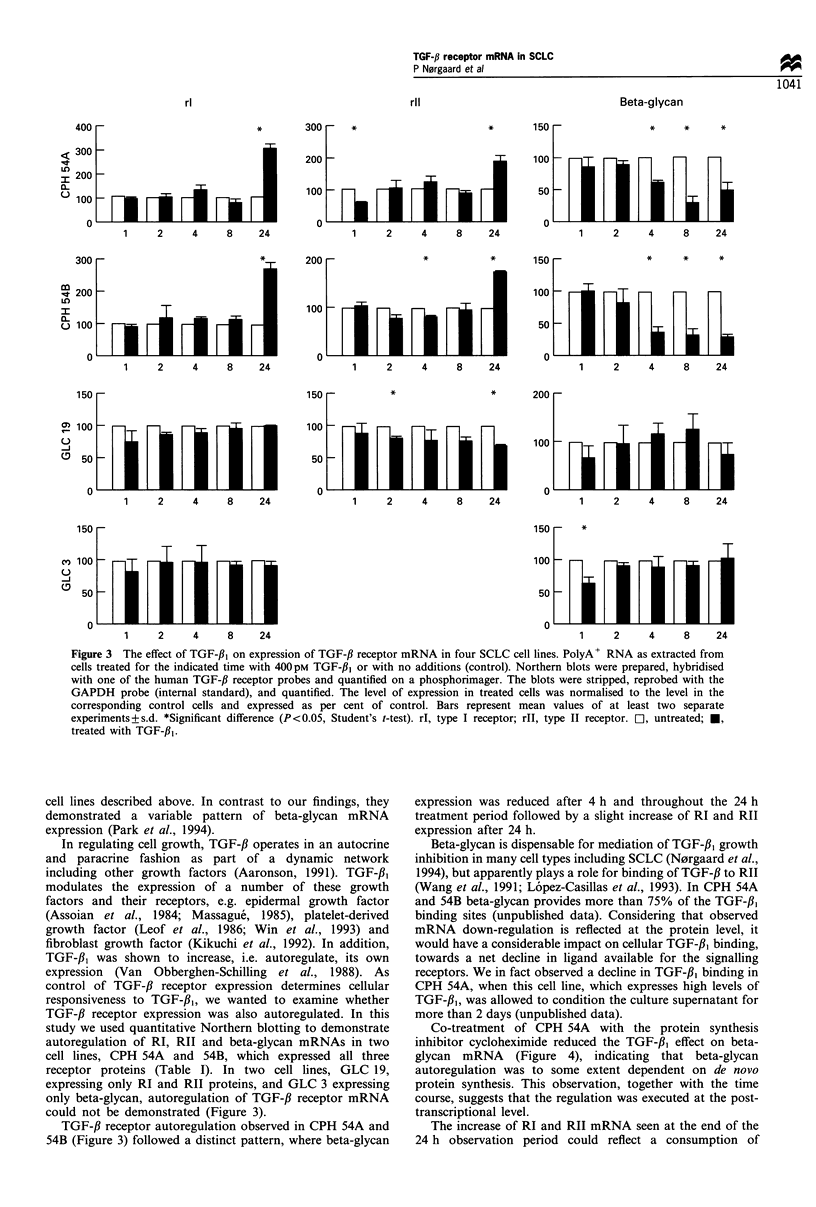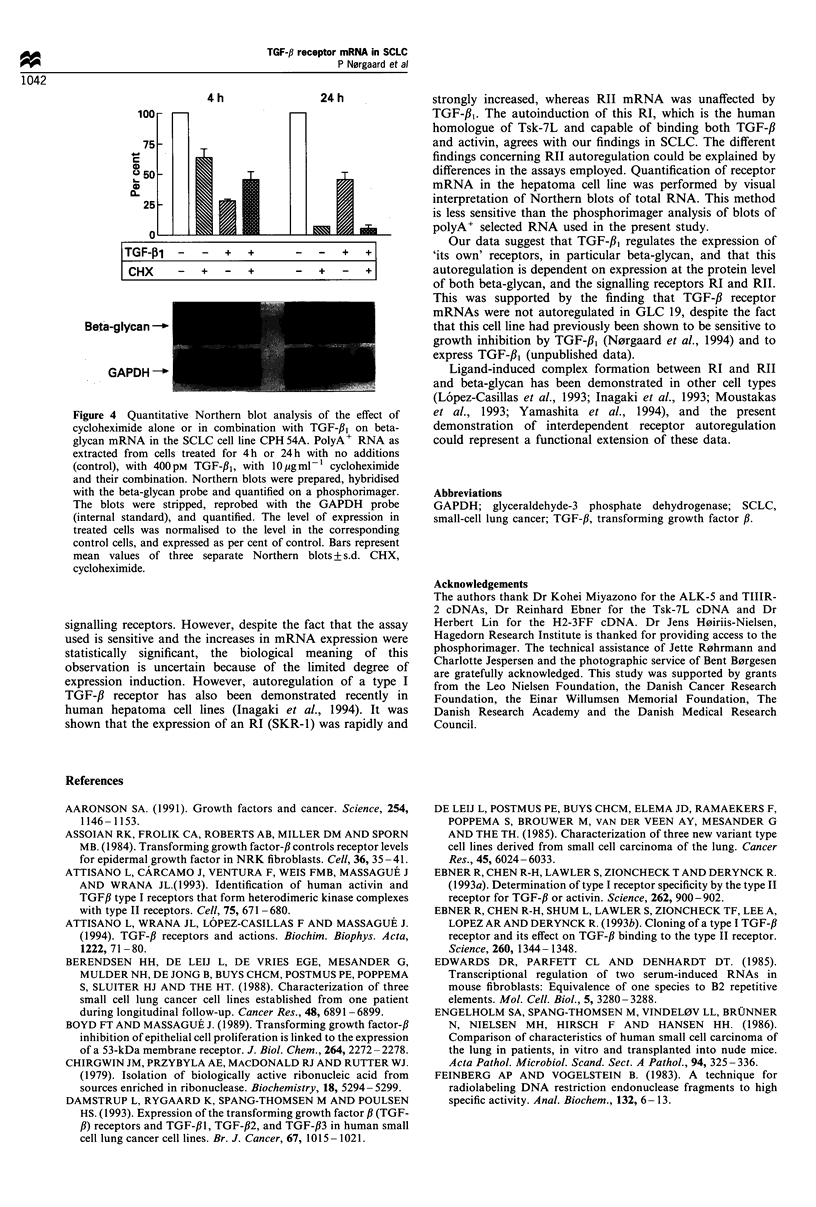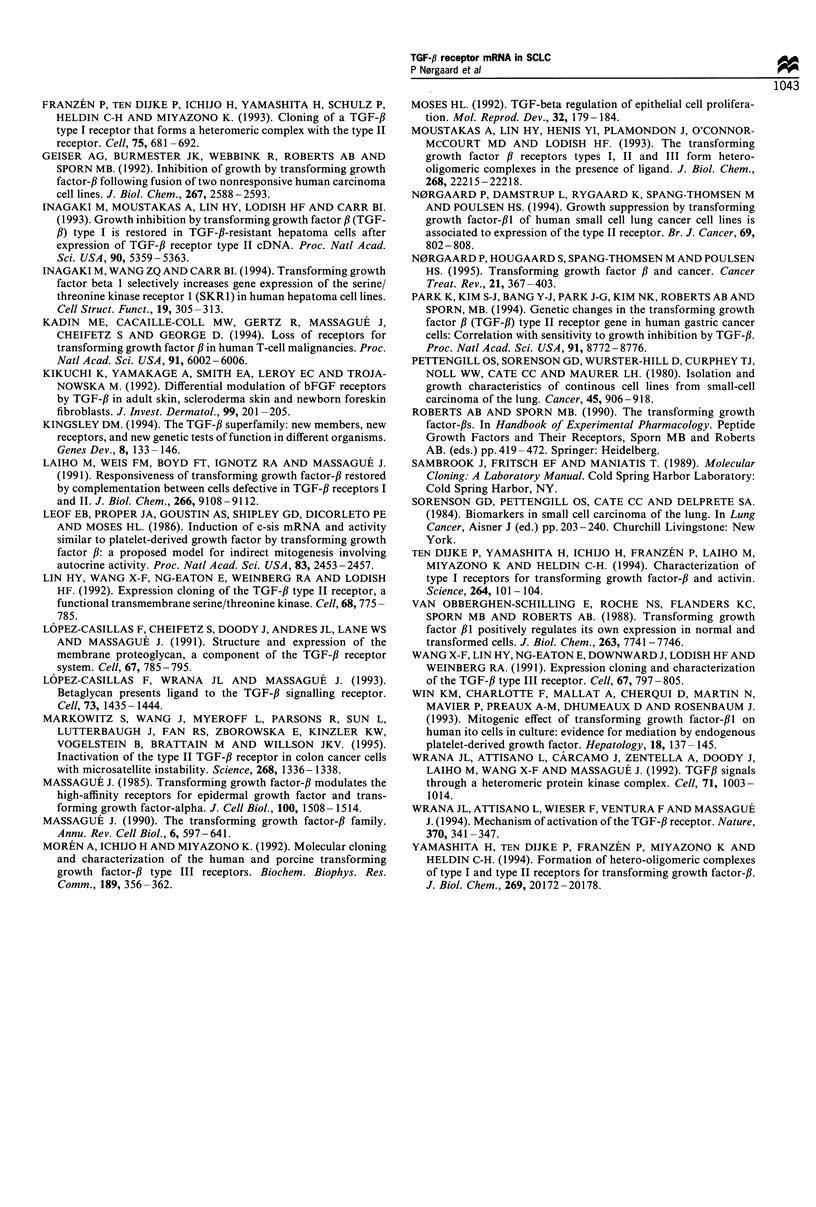# Expression and autoregulation of transforming growth factor beta receptor mRNA in small-cell lung cancer cell lines.

**DOI:** 10.1038/bjc.1996.201

**Published:** 1996-05

**Authors:** P. Nørgaard, M. Spang-Thomsen, H. S. Poulsen

**Affiliations:** Section for Radiation Biology, Finsen Center, Rigshospitalet, Copenhagen, Denmark.

## Abstract

**Images:**


British Journal of Cancer (1996) 73, 1037-1043
© 1996 Stockton Press All rights reserved 0007-0920/96 $12.00

### Expression and autoregulation of transforming growth factor $\beta$ receptor mRNA in small-cell lung cancer cell lines

P Nørgaard[1,2] M Spang-Thomsen[2] and HS Poulsen[1]

[1]*Section for Radiation Biology, The Finsen Center, Rigshospitalet, DK-2100, Copenhagen, Denmark;* [2]*Institute of Pathological Anatomy, University of Copenhagen, Frederik V's Vej 11, PO Box 2713, DK-2100 Copenhagen, Denmark.*

**Summary** In small-cell lung cancer cell lines resistance to growth inhibition by transforming growth factor (TGF)-$\beta_1$ was previously shown to correlate with lack of TGF-$\beta$ receptor I (RI) and II (RII) proteins. To further investigate the role of these receptors, the expression of mRNA for RI, RII and beta-glycan (RIII) was examined. The results showed that loss of RII mRNA correlated with TGF-$\beta_1$ resistance. In contrast, RI- and beta-glycan mRNA was expressed by all cell lines, including those lacking expression of these proteins. According to Southern blot analysis, the loss of type II mRNA was not due to gross structural changes in the gene. The effect of TGF-$\beta_1$ on expression of TGF-$\beta$ receptor mRNA (receptor autoregulation) was examined by quantitative Northern blotting in four cell lines with different expression of TGF-$\beta$ receptor proteins. In two cell lines expressing all three TGF-$\beta$ receptor proteins beta-glycan mRNA was rapidly down-regulated and this effect was sustained throughout the 24 h observation period. RI and RII mRNAs were slightly increased 24 h after treatment. In one cell line sensitive to growth inhibition by TGF-$\beta_1$, but lacking beta-glycan expression, and one cell line expressing only beta-glycan and thus TGF-$\beta_1$-resistant, no autoregulation of mRNA of either TGF-$\beta$ receptor was demonstrated. The results suggest that TGF-$\beta_1$ regulates the expression of its receptors, in particular beta-glycan, and that this effect is dependent on co-expression of beta-glycan, RI and RII.

**Keywords:** small-cell lung cancer; cell line; transforming growth factor beta receptor; transforming growth factor beta receptor mRNA; autoregulation

Transforming growth factor $\beta$ (TGF-$\beta$) is the prototype polypeptide growth factor of the TGF-$\beta$ superfamily, which also includes e.g. the activins, inhibins and bone morphogenetic protein (Massagué, 1990). TGF-$\beta$ acts in a paracrine and autocrine fashion as a multifunctional growth factor, influencing basic cellular functions such as proliferation, differentiation, cell-cell and cell-matrix interaction (Roberts and Sporn, 1990; Moses, 1992; Kingsley, 1994). Three isoforms TGF-$\beta_1$, TGF-$\beta_2$ and TGF-$\beta_3$, are expressed by many human cell types; TGF-$\beta_1$ is the most widely studied.

In mammalian cells the TGF-$\beta$ isoforms bind with high affinity and specificity to three transmembrane receptors (type I, II and beta-glycan), which are expressed by a wide range of normal and malignant cells (Attisano *et al.*, 1994; Nørgaard *et al.*, 1995).

Type I (RI) and type II (RII) receptors are structurally analogous glycoproteins with an intracellular serine/threonine kinase domain. They are considered to be the signal-transducing TGF-$\beta$ receptors, as both receptors are required for mediating the various effects of TGF-$\beta$ (Wrana *et al.*, 1992, 1994).

TGF-$\beta_1$ has a strong growth inhibitive effect *in vitro* on most ectodermally derived cell types (Nørgaard *et al.*, 1995). In small-cell lung cancer (SCLC) cell lines, resistance to growth inhibition by TGF-$\beta_1$ correlated with lack of RI and RII protein (Nørgaard *et al.*, 1994), and in other human cancer cell lines with decreased or loss of RII protein expression (Geiser *et al.*, 1992; Inagaki *et al.*, 1993; Park *et al.*, 1994). Very recently it was described that in colon cancer cell lines loss of RII mRNA and protein owing to mutations within small repeated sequences of the RII gene correlated with DNA repair defects (Markowitz *et al.*, 1995). The role of RI expression is less defined due to the fact that this receptor only binds TGF-$\beta$ in the presence of RII (Laiho *et al.*, 1991; Inagaki *et al.*, 1993). Hence, RI cannot be detected by ligand-binding assays (e.g. chemical cross-linking assay) unless co-expressed with RII. Recently, cDNAs for human RI and RII were cloned. Several different human RI receptor cDNAs were identified (Attisano *et al.*, 1993; Franzén *et al.*, 1993; ten Dijke *et al.*, 1994). Some of these had the capacity to bind the different members of the TGF-$\beta$ superfamily, depending on the type of RII with which they were co-expressed (Ebner *et al.*, 1993*a*; Attisano *et al.*, 1993; ten Dijke *et al.*, 1994). However only one RI, termed ALK-5, has been shown to respond to TGF-$\beta_1$ binding with serine/threonine kinase activity and to restore TGF-$\beta_1$ response upon transfection into RI-defective cells (Franzén *et al.*, 1993). In contrast to RI, only one RII has been found (Lin *et al.*, 1992).

Beta-glycan, also termed type III TGF-$\beta$ receptor, is a proteoglycan, which is structurally different from the type I and II receptors. It has a relatively large extracellular part, and a short cytoplasmic part, which apparently lacks a catalytic domain (López-Casillas *et al.*, 1991; Wang *et al.*, 1991; Morén *et al.*, 1992). Accordingly several cell types are responsive to TGF-$\beta$ despite lacking beta-glycan (Wang *et al.*, 1991; Boyd and Massagué, 1989; Nørgaard *et al.*, 1994; López-Casillas *et al.*, 1993), but in some cases the presence of beta-glycan in these cells increased the binding of TGF-$\beta$ to RII. Beta-glycan is therefore thought to function as a ligand capacitor, regulating the amount of ligand available for the signalling receptors RI and RII.

TGF-$\beta_1$ may exert its growth-inhibitory effect in part by regulating the expression of other polypeptide growth factors and their receptors (Assoian *et al.*, 1984; Massagué, 1985). TGF-$\beta_1$ was also shown to induce its own expression (Van Obberghen-Schilling *et al.*, 1988), but whether the expression of TGF-$\beta$ receptors is regulated by TGF-$\beta_1$ (autoregulation) has not yet been described.

In this study we characterised the expression of mRNA for RI (ALK-5) and RII and beta-glycan in a panel of nine SCLC cell lines previously examined for TGF-$\beta_1$ sensitivity (Nørgaard *et al.*, 1994) and with different expression of TGF-$\beta$ receptor proteins (Damstrup *et al.*, 1993). In addition, we studied autoregulation of TGF-$\beta$ receptor mRNA in four of the cell lines, by quantitating the effect of exogenous TGF-$\beta_1$ on the expression of these mRNAs.

Correspondence: HS Poulsen
Received 25 August 1995; revised 13 November 1995; accepted 4 December 1995

The results showed an unequivocal correlation between expression of RII mRNA and sensitivity to growth inhibition by TGF-$\beta_1$, whereas all cell lines expressed RI and beta-glycan mRNA, regardless of whether these receptor proteins were expressed. According to Southern blot analysis lack of RII mRNA was not due to gross structural changes in the gene encoding this receptor. TGF-$\beta$ receptor mRNA was autoregulated in two cell lines expressing all three receptor proteins. Beta-glycan mRNA was rapidly down-regulated, whereas RI and RII mRNAs were subsequently increased 2- to 3-fold. In one cell line expressing RI and RII proteins but not beta-glycan, and thus still sensitive to growth inhibition by TGF-$\beta_1$, and one TGF-$\beta_1$-resistant cell line expressing only beta-glycan, TGF-$\beta$ receptor mRNA expressions were practically unaffected by TGF-$\beta_1$. Autoregulation of beta-glycan mRNA autoregulation was partially antagonised by addition of cycloheximide, indicating that *de novo* protein synthesis was to some extent required for this effect. The results suggest that TGF-$\beta$ receptor autoregulation is dependent on expression of an 'intact' receptor system, and thus provides an example of a functional interaction between RI, RII and beta-glycan, not previously described.

### Materials and methods

### *Cell lines*

SCLC cell lines were cultured in 525 $cm^2$ triple flasks (NUNC) at 37°C, 5% carbon dioxide and 80% humidity in medium containing 10% inactivated (56°C, 30 min) fetal calf serum (Flow Laboratories, Irvine, UK) without antibiotics. Nine SCLC cell lines established from six patients, and characterised as SCLC cell lines in three different laboratories, were examined. Four cell lines established at Dartmouth Medical School, Hanover, NH, USA (DMS 53, DMS 92, DMS 114, DMS 273) (Pettengill *et al.*, 1980; Sorenson *et al.*, 1984) were cultured in Waymouth medium (Gibco, Paisley, UK). Three cell lines established at Groningen Lung Cancer Center, Groningen, The Netherlands (GLC 3, GLC 16, GLC 19) (Berendsen *et al.*, 1988; De Leij *et al.*, 1985) were cultured in RPMI-1640 (Gibco) and two cell lines established in our laboratory (CPH 54A, CPH 54B) (Engelholm *et al.*, 1986) were grown in EMEM (Eagle's minimum essential medium) (Gibco). The cells were passaged twice a week. Cells growing as monolayer cultures (CPH 54A, CPH 54B, DMS 53, DMS 92, DMS 114, DMS 273) were passaged with trypsin. Cells growing as floating aggregates (GLC 3, GLC 16, GLC 19) were allowed to sediment before replacing the medium.

All cell lines were routinely checked for, and found free of, mycoplasma infection.

For steady-state mRNA expression studies subconfluent, exponentially growing cells were harvested using a cell scraper and centrifuged at 1100 ***g***. The cell pellet was frozen immediately in liquid nitrogen and stored at −70°C until analysis.

### *Growth factor treatment assay*

Porcine TGF-$\beta_1$ was purchased from R&D Systems Europe, Oxon, UK. Dried TGF-$\beta_1$ (1 $\mu$g) was reconstitued in 0.5 ml of 4 mM hydrochloric acid containing 2 mg $ml^{-1}$ bovine serum albumin and stored at 4°C. For treatment assays, culture medium (with 10% fetal calf serum) containing 400 pM TGF-$\beta_1$ was made immediately before each experiment.

Exponentially growing cells were harvested as described above, resuspended in PBS and centrifuged at 275 ***g*** for 5 min. Single cell suspension was obtained by mechanical disaggregation. Cells were counted in a haemocytometer and viability was evaluated by the trypan blue exclusion test. Approximately $5 \times 10^6$ viable cells were seeded in 175 $cm^2$ culture flasks.

Cells were treated with TGF-$\beta_1$ as previously described (Nørgaard *et al.*, 1994). Briefly, cells growing as monolayer cultures were allowed to attach for 24 h. Culture medium was removed from the subconfluent cells and prewarmed (37°C) medium $\pm$ 400 pM TGF-$\beta_1$ was added (designated $T=0$ h). Cells growing as floating aggregates were seeded directly in prewarmed (37°C) medium $\pm$ 400 pM TGF-$\beta_1$ ($T=0$ h). After 1, 2, 4, 8 and 24 h treated cells and untreated cells (control) were harvested as described above. All experiments were reproduced at least twice.

### *Cycloheximide treatment*

Where indicated, 10 $\mu$g $ml^{-1}$ cycloheximide (Sigma, St Louis, MO, USA) dissolved in PBS was added to the culture medium alone or in combination with TGF-$\beta_1$, and cells were harvested as described above after 4 and 24 h.

### *RNA extraction, electrophoresis and blotting*

Poly$A^+$ RNA as extracted directly from the frozen (−70°C) cell pellets by the guanidinium thiocyanate method followed by two sequential purifications by oligo(dT)-cellulose chromatography using a commercial kit (QuickPrep mRNA Purification Kit, Pharmacia) (Chirgwin *et al.*, 1979). The concentration was determined by spectrophotometry, and the RNA was precipitated under ethanol at −70°C, in aliquots of 3−5 $\mu$g. The RNA was dissolved in sample buffer (50% formamide, 2.2 M formaldehyde, 20 mM 3-morpholino-propane-sulphonic acid, 5 mM sodium acetate, 1 mM EDTA, 2% Ficoll 400, 0.25% bromophenol blue) containing 0.033 $\mu$g $\mu l^{-1}$ ethidium bromide and electrophoresed under denaturing conditions in 1% agarose gels containing 2.2 M formaldehyde, together with 5 $\mu$g RNA size marker (RNA ladder, Gibco BRL). Transfer to charged nylon membranes (Gene Screen Plus, NEN DuPont) was done in 10 × saline sodium citrate (1 × saline sodium citrate = 150 mM sodium chloride, 15 mM sodium citrate). The Northern blots were prehybridised for at least 3 h and hybridised for 18 h at 42°C in a buffer containing 50% formamide, 1% sodium dodecylsulphate, 1 M sodium chloride, 5% dextran sulphate, 100 $\mu$g $ml^{-1}$ denatured salmon testes DNA. Maximal washing stringency was 65°C in 2 × saline sodium citrate, 1% sodium dodecylsulphate.

For determination of steady-state mRNA expression Northern blots were exposed to X-ray film (Amersham) at −80°C with an intensifying screen for 1−7 days. The blots were hybridised and rehybridised with the three TGF-$\beta$ receptor probes, followed by the glyceraldehyde-3 phosphate dehydrogenase (GAPDH) probe, which was used as an internal standard to compare the amounts of poly$A^+$ RNA transferred to the membranes.

### *RNA quantification*

In order to minimise loss of mRNA from the membranes during the high stringency wash performed to strip off the probes, Northern blots for quantitative measurements were used for only one hybridisation with a TGF-$\beta$ receptor probe and subsequently reprobed with the GAPDH standard probe. The hybridised Northern blots were analysed on a phosphorimager (Molecular Dynamics). The amounts of receptor mRNA were normalised against the corresponding amounts of GAPDH mRNA, which was found to be unaffected by TGF-$\beta_1$ (Edwards *et al.*, 1985). The normalised amounts of TGF-$\beta$ receptor mRNA in cells treated with TGF-$\beta_1$ were expressed relative to the respective mRNA amounts of the corresponding untreated cells (per cent of control). The differences in expression levels were evaluated by the Student's *t*-test (significance level; $P<0.05$). After phosphorimager analysis autoradiography of the blots as performed as described above, with the purpose of photo reproduction.

*DNA extraction, electrophoresis and blotting*

DNA was extracted with phenol and chloroform by standard methods (Sambrook *et al.*, 1989). Digestion with restriction endonucleases *Bam*HI or *Pst*I was performed as recommended by the supplier (Gibco BRL, Life Technologies Europe). Aliquots of 10 μg/lane were electrophoresed in 0.8% agarose gels and transferred to charged nylone membranes (Gene Screen Plus, NEN DuPont). Prehybridisation, hybridisation and washing were as recommended by the supplier: washing stringency was 2 × saline sodium citrate–1% sodium dodecylsulphate twice for 30 min at 60°C. Southern blots were reprobed with a human GAPDH probe. Membranes were exposed to X-ray film (Amersham) at −80°C with an intensifying screen for 7 days.

*Probes*

Two RI probes were used. One was a 960 bp *Eco*RI fragment of the cloned 2.3 kb ALK-5 human cDNA kindly provided by K Miyazono (Franzén *et al.*, 1993). The other was the full length (2.4 kb *Eco*RI insert) Tsk-7L murine cDNA kindly provided by R Ebner (Ebner *et al.*, 1993*b*). The RII probe was a 930 bp *Pst*I-*Sac*I fragment of the cloned 4.5 kb H2-3FF human cDNA kindly provided by HY Lin (Lin *et al.*, 1992). The probe for beta-glycan was a 1.1 kb *Pst*I fragment of the cloned 4.2 kb TIIIR-2 human cDNA kindly provided by K Miyazono (Morén *et al.*, 1992). The human GAPDH probe was a 1.1 kb cDNA fragment purchased from Clontech (Palo Alto, CA, USA), hybridising to a ~1.4 kb mRNA. Isolation of cDNA probes was done by standard methods (Sambrook *et al.*, 1989). Radiolabelled probes were prepared by the random priming method (Feinberg and Vogelstein, 1983) using a commercial kit and [α-$^{32}$P]dCTP (both from Amersham).

### Results

*Steady-state TGF-β receptor mRNA expression*

The steady-state expressions of TGF-β receptor mRNA of the examined SCLC cell lines were analysed by Northern blotting. All nine cell lines expressed a single ~6.5 kb RI mRNA (ALK-5) and a single ~7.0 kb mRNA for beta-glycan, whereas only six cell lines expressed a ~5.5 kb RII mRNA (Figure 1).

*Southern blotting*

The SCLC cell lines were examined for gross structural DNA changes of the RII gene. Southern blots of genomic DNA digested with *Bam*HI or *Pst*I were hybridised with the RII probe. No absent or abnormal bands were detected in the three SCLC cell lines lacking RII mRNA expression (DMS 114, DMS 92, GLC 3) compared with the six cell lines expressing RII mRNA (data not shown).

*Effect of TGF-$\beta_1$ on TGF-β receptor mRNA levels*

The effect of TGF-$\beta_1$ on the expression of TGF-β receptor mRNA over a 24 h period was examined by quantitative Northern blot analysis in four SCLC cell lines with different TGF-$\beta_1$ sensitivity and receptor protein expression: CPH 54A and CPH 54B, GLC 19, GLC 3.

CPH 54A and 54B express all three TGF-β receptors at the mRNA and protein levels (Figure 1 and Table I). Treatment of these cell lines with TGF-$\beta_1$ resulted in a statistically significant increase in expression of RI and RII mRNA detected after 24 h of treatment (Figures 2 and 3). RI mRNA was increased approximately 3-fold, whereas RII mRNA was increased about 2-fold compared with control levels. The responses were very similar in the two cell lines. CPH 54A and 54B also expressed mRNA for another RI, Tsk-7L (Ebner *et al.*, 1993*b*), which is the murine homologue

**Figure 1** Northern blot anlaysis of TGF-β receptor I, II and beta-glycan mRNA in nine SCLC cell lines. PolyA$^+$ RNA (3–5 μl/lane) were electrophoresed in formaldehyde gel, transferred to nylon membranes and subsequently hybridised with the human TGF-β receptor probes: type I (ALK-5), type II (H2-3FF), beta-glycan (TIIIR-2). The membranes were reprobed with the GAPDH probe, as an internal standard, to compare the amount of RNA transferred to the membranes. rI, type I receptor; rII, type II receptor.

**Table 1** Expression of TGF-β receptor type I, II and beta-glycan mRNA and protein, and *in vitro* sensitivity to growth inhibition by TGF-$\beta_1$, in nine SCLC cell lines

| | TGF-β-r mRNA | | | TGF-β-r protein[a] | | | TGF-$\beta_1$ growth inhibition[b] |
|---|---|---|---|---|---|---|---|
| Cell line | I | II | BG | I | II | BG | |
| CPH 54A | + | + | + | + | + | + | + |
| CPH 54B | + | + | + | + | + | + | + |
| GLC 16 | + | + | + | + | + | − | + |
| GLC 19 | + | + | + | + | + | − | + |
| DMS 273 | + | + | + | + | (+) | + | + |
| DMS 53 | + | + | + | − | − | − | (+) |
| DMS 114 | + | − | + | − | − | + | − |
| GLC 3 | + | − | + | − | − | + | − |
| DMS 92 | + | − | + | − | − | − | NT |

[a]Examined by chemical cross-linking assay. Data from Damstrup *et al.* (1993). [b]Data from Nørgaard *et al.* (1994). BG, beta-glycan (type III receptor); NT, not tested. Parenthesis indicates weak type II receptor expression and weak growth inhibition respectively.

**Figure 2** Northern blot analysis of TGF-$\beta$ receptor I, II and beta-glycan mRNA in the SCLC cell line CPH 54B treated with TGF-$\beta_1$. The cells were grown for the indicated time in the presence (+) or absence (−) of 400 pM TGF-$\beta_1$. PolyA$^+$ RNA (3–5 $\mu$g/lane) were electrophoresed in formaldehyde gel, transferred to nylon membranes, and hybridised with one of the human TGF-$\beta$ receptor probes: type I (ALK-5), type II (H2-3FF), beta-glycan (TIIIR-2). The membranes were stripped and reprobed with the GAPDH probe (internal standard), to compare the amount of RNA transferred to the membranes. rI, type I receptor; rII, type II receptor.

of the human ALK-2 receptor. Tsk-7L cDNA, which hybridised to a ~3.3 kb mRNA, was increased by TGF-$\beta_1$ in a manner similar to ALK-5, i.e. 3-fold increase after 24 h treatment (data not shown).

In contrast to RI and RII beta-glycan mRNA in CPH 54A and 54B was reduced by TGF-$\beta_1$ (Figures 2 and 3). The reduction 4 h after treatment was more pronounced in CPH 54B than in 54A, but in both cell lines maximal inhibition was 30–40% of the level of untreated cells. The inhibition of beta-glycan mRNA was sustained throughout the 24 h observation period (Figures 2 and 3).

GLC 19 lacks beta-glycan expression at the protein level, but expresses mRNA for all three receptors, and GLC 3 expresses only beta-glycan at the protein level (Table I). When these cells were treated with TGF-$\beta_1$, no statistically significant modulation of TGF-$\beta$ receptor mRNA was observed. In GLC 19 RII mRNA showed a slight decreasing tendency, which was, however, not statistically significant throughout the experiment (Figure 3). As indicated with asterisks in Figure 3 minor changes in RII mRNA expression in CPH 54A, 54B and GLC 19 were observed during the first 8 h, and a slight decrease in beta-glycan expression was seen in GLC 3. The limited magnitude and extension of these changes, however, suggest that they represent methodological artefacts.

*Effect of cycloheximide on beta-glycan mRNA level*

To test whether protein synthesis was required for the observed down-regulation of the beta-glycan mRNA level (Figure 3), CPH 54A was incubated for 4 or 24 h with cycloheximide (10 $\mu$g ml$^{-1}$) alone or in combination with 400 pM TGF-$\beta_1$. Quantitative Northern blot analysis showed that cycloheximide by itself reduced beta-glycan mRNA, but in a less pronounced way than TGF-$\beta_1$ after 4 h treatment (Figure 4). Four hours' cycloheximide treatment antagonised the TGF-$\beta_1$ effect. After 24 h cycloheximide markedly reduced beta-glycan mRNA both alone and in combination with TGF-$\beta_1$.

### Discussion

We previously found that sensitivity to the growth-inhibitory effect of TGF-$\beta_1$ correlated with expression of RI and RII proteins, in a panel of nine SCLC cell lines (Nørgaard *et al.*, 1994). TGF-$\beta$ receptors in these cell lines were determined by chemical cross-linking of radiolabelled TGF-$\beta_1$ (Damstrup *et al.*, 1993), and as RI only binds TGF-$\beta_1$ when co-expressed with RII (Laiho *et al.*, 1991; Inagaki *et al.*, 1993), these data could not exclude the possibility that some of the 'RII-negative' cell lines in fact expressed RI.

We therefore studied the expression of mRNA for the TGF-$\beta$ receptors in the panel of SCLC cell lines, and found a correlation between expression of RII mRNA and sensitivity to TGF-$\beta_1$ (Table I). RI and beta-glycan mRNAs were expressed by all cell lines examined, including the cell lines in which these receptor proteins were not detected (Table I), thus there was no correlation between expression of RI or beta-glycan mRNA and sensitivity to growth inhibition by TGF-$\beta_1$ (Table I). These results emphasise the role of RII as determinant for TGF-$\beta_1$-sensitivity in SCLC. There was a correlation between expression of RII mRNA and protein except for one cell line, DMS 53, in which the protein was not detected (Damstrup *et al.*, 1993) (Table I). DMS 53 was earlier found to respond weakly to TGF-$\beta_1$, and we concluded that RII protein was expressed below the detection limit of the chemical cross-linking assay (Nørgaard *et al.*, 1994).

Previously only three reports have described the genetic background for loss of RII in human cancer cells. In one study a panel of colon cancer cell lines was found to harbour mutations in the RII gene, resulting in absent or low levels of RII transcripts and resistance to TGF-$\beta$ growth inhibition (Markowitz *et al.*, 1995). There was a highly significant correlation between escape from the growth-inhibitive effect of TGF-$\beta$ and microsatellite instability resulting in DNA repair defects. In gastric cancer cell lines (Park *et al.*, 1994) and human T-cell malignancy (Kadih *et al.*, 1994) TGF-$\beta$ resistance was likewise due to altered RII mRNA, caused by amplifications or deletions of the RII gene. Our demonstration of lack of RII mRNA as the cause of TGF-$\beta_1$ resistance in SCLC, together with the finding that 16/21 SCLC cell lines lack RI and RII proteins (Damstrup *et al.*, 1993), add to an emerging picture of a frequent defect of importance in growth regulation in cancer. In agreement with this, we found no signs of deletion or translocation of the RII gene by Southern blotting (data not shown), indicating that the lack of RII mRNA in SCLC could be due to point mutations or silenced transcription.

For RI and beta-glycan we demonstrated a discrepancy between mRNA and protein expression (Table I). This opens the possibility that RI protein was in fact expressed by the 'RII-negative' cell lines, but inaccessible to detection by the chemical cross-linking assay, as earlier demonstrated in human hepatoma cell lines (Inagaki *et al.*, 1993) and mink lung cells (Laiho *et al.*, 1991). Four cell lines expressed beta-glycan mRNA despite lack of beta-glycan protein expression (Table I). We could conclude that no deletion or translocation of the beta-glycan gene had occurred in these cell lines, since they all expressed a ~7.0 kb transcript (Figure 1), and Southern blot analysis showed no evidence of structural changes in the gene (data not shown). The only previous characterisation of beta-glycan mRNA expression in human cancer cells was done on the human gastric carcinoma

**Figure 3** The effect of TGF-$\beta_1$ on expression of TGF-$\beta$ receptor mRNA in four SCLC cell lines. PolyA$^+$ RNA as extracted from cells treated for the indicated time with 400 pM TGF-$\beta_1$ or with no additions (control). Northern blots were prepared, hybridised with one of the human TGF-$\beta$ receptor probes and quantified on a phosphorimager. The blots were stripped, reprobed with the GAPDH probe (internal standard), and quantified. The level of expression in treated cells was normalised to the level in the corresponding control cells and expressed as per cent of control. Bars represent mean values of at least two separate experiments ± s.d. *Significant difference ($P < 0.05$, Student's $t$-test). rI, type I receptor; rII, type II receptor. □, untreated; ■, treated with TGF-$\beta_1$.

cell lines described above. In contrast to our findings, they demonstrated a variable pattern of beta-glycan mRNA expression (Park *et al.*, 1994).

In regulating cell growth, TGF-$\beta$ operates in an autocrine and paracrine fashion as part of a dynamic network including other growth factors (Aaronson, 1991). TGF-$\beta_1$ modulates the expression of a number of these growth factors and their receptors, e.g. epidermal growth factor (Assoian *et al.*, 1984; Massagué, 1985), platelet-derived growth factor (Leof *et al.*, 1986; Win *et al.*, 1993) and fibroblast growth factor (Kikuchi *et al.*, 1992). In addition, TGF-$\beta_1$ was shown to increase, i.e. autoregulate, its own expression (Van Obberghen-Schilling *et al.*, 1988). As control of TGF-$\beta$ receptor expression determines cellular responsiveness to TGF-$\beta_1$, we wanted to examine whether TGF-$\beta$ receptor expression was also autoregulated. In this study we used quantitative Northern blotting to demonstrate autoregulation of RI, RII and beta-glycan mRNAs in two cell lines, CPH 54A and 54B, which expressed all three receptor proteins (Table I). In two cell lines, GLC 19, expressing only RI and RII proteins, and GLC 3 expressing only beta-glycan, autoregulation of TGF-$\beta$ receptor mRNA could not be demonstrated (Figure 3).

TGF-$\beta$ receptor autoregulation observed in CPH 54A and 54B (Figure 3) followed a distinct pattern, where beta-glycan expression was reduced after 4 h and throughout the 24 h treatment period followed by a slight increase of RI and RII expression after 24 h.

Beta-glycan is dispensable for mediation of TGF-$\beta_1$ growth inhibition in many cell types including SCLC (Nørgaard *et al.*, 1994), but apparently plays a role for binding of TGF-$\beta$ to RII (Wang *et al.*, 1991; López-Casillas *et al.*, 1993). In CPH 54A and 54B beta-glycan provides more than 75% of the TGF-$\beta_1$ binding sites (unpublished data). Considering that observed mRNA down-regulation is reflected at the protein level, it would have a considerable impact on cellular TGF-$\beta_1$ binding, towards a net decline in ligand available for the signalling receptors. We in fact observed a decline in TGF-$\beta_1$ binding in CPH 54A, when this cell line, which expresses high levels of TGF-$\beta_1$, was allowed to condition the culture supernatant for more than 2 days (unpublished data).

Co-treatment of CPH 54A with the protein synthesis inhibitor cycloheximide reduced the TGF-$\beta_1$ effect on beta-glycan mRNA (Figure 4), indicating that beta-glycan autoregulation was to some extent dependent on *de novo* protein synthesis. This observation, together with the time course, suggests that the regulation was executed at the post-transcriptional level.

The increase of RI and RII mRNA seen at the end of the 24 h observation period could reflect a consumption of

**Figure 4** Quantitative Northern blot analysis of the effect of cycloheximide alone or in combination with TGF-$\beta_1$ on beta-glycan mRNA in the SCLC cell line CPH 54A. PolyA$^+$ RNA as extracted from cells treated for 4 h or 24 h with no additions (control), with 400 pM TGF-$\beta_1$, with 10 $\mu$g ml$^{-1}$ cycloheximide and their combination. Northern blots were prepared, hybridised with the beta-glycan probe and quantified on a phosphorimager. The blots were stripped, reprobed with the GAPDH probe (internal standard), and quantified. The level of expression in treated cells was normalised to the level in the corresponding control cells, and expressed as per cent of control. Bars represent mean values of three separate Northern blots±s.d. CHX, cycloheximide.

signalling receptors. However, despite the fact that the assay used is sensitive and the increases in mRNA expression were statistically significant, the biological meaning of this observation is uncertain because of the limited degree of expression induction. However, autoregulation of a type I TGF-β receptor has also been demonstrated recently in human hepatoma cell lines (Inagaki *et al.*, 1994). It was shown that the expression of an RI (SKR-1) was rapidly and strongly increased, whereas RII mRNA was unaffected by TGF-$\beta_1$. The autoinduction of this RI, which is the human homologue of Tsk-7L and capable of binding both TGF-β and activin, agrees with our findings in SCLC. The different findings concerning RII autoregulation could be explained by differences in the assays employed. Quantification of receptor mRNA in the hepatoma cell line was performed by visual interpretation of Northern blots of total RNA. This method is less sensitive than the phosphorimager analysis of blots of polyA$^+$ selected RNA used in the present study.

Our data suggest that TGF-$\beta_1$ regulates the expression of 'its own' receptors, in particular beta-glycan, and that this autoregulation is dependent on expression at the protein level of both beta-glycan, and the signalling receptors RI and RII. This was supported by the finding that TGF-β receptor mRNAs were not autoregulated in GLC 19, despite the fact that this cell line had previously been shown to be sensitive to growth inhibition by TGF-$\beta_1$ (Nørgaard *et al.*, 1994) and to express TGF-$\beta_1$ (unpublished data).

Ligand-induced complex formation between RI and RII and beta-glycan has been demonstrated in other cell types (López-Casillas *et al.*, 1993; Inagaki *et al.*, 1993; Moustakas *et al.*, 1993; Yamashita *et al.*, 1994), and the present demonstration of interdependent receptor autoregulation could represent a functional extension of these data.

### Abbreviations

GAPDH; glyceraldehyde-3 phosphate dehydrogenase; SCLC, small-cell lung cancer; TGF-β, transforming growth factor β.

### Acknowledgements

The authors thank Dr Kohei Miyazono for the ALK-5 and TIIIR-2 cDNAs, Dr Reinhard Ebner for the Tsk-7L cDNA and Dr Herbert Lin for the H2-3FF cDNA. Dr Jens Høiriis-Nielsen, Hagedorn Research Institute is thanked for providing access to the phosphorimager. The technical assistance of Jette Røhrmann and Charlotte Jespersen and the photographic service of Bent Børgesen are gratefully acknowledged. This study was supported by grants from the Leo Nielsen Foundation, the Danish Cancer Research Foundation, the Einar Willumsen Memorial Foundation, The Danish Research Academy and the Danish Medical Research Council.

### References

AARONSON SA. (1991). Growth factors and cancer. *Science*, **254**, 1146–1153.

ASSOIAN RK, FROLIK CA, ROBERTS AB, MILLER DM AND SPORN MB. (1984). Transforming growth factor-β controls receptor levels for epidermal growth factor in NRK fibroblasts. *Cell*, **36**, 35–41.

ATTISANO L, CÁRCAMO J, VENTURA F, WEIS FMB, MASSAGUÉ J AND WRANA JL.(1993). Identification of human activin and TGFβ type I receptors that form heterodimeric kinase complexes with type II receptors. *Cell*, **75**, 671–680.

ATTISANO L, WRANA JL, LÓPEZ-CASILLAS F AND MASSAGUÉ J. (1994). TGF-β receptors and actions. *Biochim. Biophys. Acta*, **1222**, 71–80.

BERENDSEN HH, DE LEIJ L, DE VRIES EGE, MESANDER G, MULDER NH, DE JONG B, BUYS CHCM, POSTMUS PE, POPPEMA S, SLUITER HJ AND THE HT. (1988). Characterization of three small cell lung cancer cell lines established from one patient during longitudinal follow-up. *Cancer Res.*, **48**, 6891–6899.

BOYD FT AND MASSAGUÉ J. (1989). Transforming growth factor-β inhibition of epithelial cell proliferation is linked to the expression of a 53-kDa membrane receptor. *J. Biol. Chem.*, **264**, 2272–2278.

CHIRGWIN JM, PRZYBYLA AE, MACDONALD RJ AND RUTTER WJ. (1979). Isolation of biologically active ribonucleic acid from sources enriched in ribonuclease. *Biochemistry*, **18**, 5294–5299.

DAMSTRUP L, RYGAARD K, SPANG-THOMSEN M AND POULSEN HS. (1993). Expression of the transforming growth factor β (TGF-β) receptors and TGF-β1, TGF-β2, and TGF-β3 in human small cell lung cancer cell lines. *Br. J. Cancer*, **67**, 1015–1021.

DE LEIJ L, POSTMUS PE, BUYS CHCM, ELEMA JD, RAMAEKERS F, POPPEMA S, BROUWER M, VAN DER VEEN AY, MESANDER G AND THE TH. (1985). Characterization of three new variant type cell lines derived from small cell carcinoma of the lung. *Cancer Res.*, **45**, 6024–6033.

EBNER R, CHEN R-H, LAWLER S, ZIONCHECK T AND DERYNCK R. (1993*a*). Determination of type I receptor specificity by the type II receptor for TGF-β or activin. *Science*, **262**, 900–902.

EBNER R, CHEN R-H, SHUM L, LAWLER S, ZIONCHECK TF, LEE A, LOPEZ AR AND DERYNCK R. (1993*b*). Cloning of a type I TGF-β receptor and its effect on TGF-β binding to the type II receptor. *Science*, **260**, 1344–1348.

EDWARDS DR, PARFETT CL AND DENHARDT DT. (1985). Transcriptional regulation of two serum-induced RNAs in mouse fibroblasts: Equivalence of one species to B2 repetitive elements. *Mol. Cell. Biol.*, **5**, 3280–3288.

ENGELHOLM SA, SPANG-THOMSEN M, VINDELØV LL, BRÜNNER N, NIELSEN MH, HIRSCH F AND HANSEN HH. (1986). Comparison of characteristics of human small cell carcinoma of the lung in patients, in vitro and transplanted into nude mice. *Acta Pathol. Microbiol. Scand. Sect. A Pathol.*, **94**, 325–336.

FEINBERG AP AND VOGELSTEIN B. (1983). A technique for radiolabeling DNA restriction endonuclease fragments to high specific activity. *Anal. Biochem.*, **132**, 6–13.

FRANZÉN P, TEN DIJKE P, ICHIJO H, YAMASHITA H, SCHULZ P, HELDIN C-H AND MIYAZONO K. (1993). Cloning of a TGF-β type I receptor that forms a heteromeric complex with the type II receptor. *Cell*, **75**, 681–692.

GEISER AG, BURMESTER JK, WEBBINK R, ROBERTS AB AND SPORN MB. (1992). Inhibition of growth by transforming growth factor-β following fusion of two nonresponsive human carcinoma cell lines. *J. Biol. Chem.*, **267**, 2588–2593.

INAGAKI M, MOUSTAKAS A, LIN HY, LODISH HF AND CARR BI. (1993). Growth inhibition by transforming growth factor β (TGF-β) type I is restored in TGF-β-resistant hepatoma cells after expression of TGF-β receptor type II cDNA. *Proc. Natl Acad. Sci. USA*, **90**, 5359–5363.

INAGAKI M, WANG ZQ AND CARR BI. (1994). Transforming growth factor beta 1 selectively increases gene expression of the serine/threonine kinase receptor 1 (SKR1) in human hepatoma cell lines. *Cell Struct. Funct.*, **19**, 305–313.

KADIN ME, CACAILLE-COLL MW, GERTZ R, MASSAGUÉ J, CHEIFETZ S AND GEORGE D. (1994). Loss of receptors for transforming growth factor β in human T-cell malignancies. *Proc. Natl Acad. Sci. USA*, **91**, 6002–6006.

KIKUCHI K, YAMAKAGE A, SMITH EA, LEROY EC AND TROJANOWSKA M. (1992). Differential modulation of bFGF receptors by TGF-β in adult skin, scleroderma skin and newborn foreskin fibroblasts. *J. Invest. Dermatol.*, **99**, 201–205.

KINGSLEY DM. (1994). The TGF-β superfamily: new members, new receptors, and new genetic tests of function in different organisms. *Genes Dev.*, **8**, 133–146.

LAIHO M, WEIS FM, BOYD FT, IGNOTZ RA AND MASSAGUÉ J. (1991). Responsiveness of transforming growth factor-β restored by complementation between cells defective in TGF-β receptors I and II. *J. Biol. Chem.*, **266**, 9108–9112.

LEOF EB, PROPER JA, GOUSTIN AS, SHIPLEY GD, DICORLETO PE AND MOSES HL. (1986). Induction of c-sis mRNA and activity similar to platelet-derived growth factor by transforming growth factor β: a proposed model for indirect mitogenesis involving autocrine activity. *Proc. Natl Acad. Sci. USA*, **83**, 2453–2457.

LIN HY, WANG X-F, NG-EATON E, WEINBERG RA AND LODISH HF. (1992). Expression cloning of the TGF-β type II receptor, a functional transmembrane serine/threonine kinase. *Cell*, **68**, 775–785.

LÓPEZ-CASILLAS F, CHEIFETZ S, DOODY J, ANDRES JL, LANE WS AND MASSAGUÉ J. (1991). Structure and expression of the membrane proteoglycan, a component of the TGF-β receptor system. *Cell*, **67**, 785–795.

LÓPEZ-CASILLAS F, WRANA JL AND MASSAGUÉ J. (1993). Betaglycan presents ligand to the TGF-β signalling receptor. *Cell*, **73**, 1435–1444.

MARKOWITZ S, WANG J, MYEROFF L, PARSONS R, SUN L, LUTTERBAUGH J, FAN RS, ZBOROWSKA E, KINZLER KW, VOGELSTEIN B, BRATTAIN M AND WILLSON JKV. (1995). Inactivation of the type II TGF-β receptor in colon cancer cells with microsatellite instability. *Science*, **268**, 1336–1338.

MASSAGUÉ J. (1985). Transforming growth factor-β modulates the high-affinity receptors for epidermal growth factor and transforming growth factor-alpha. *J. Cell Biol.*, **100**, 1508–1514.

MASSAGUÉ J. (1990). The transforming growth factor-β family. *Annu. Rev. Cell Biol.*, **6**, 597–641.

MORÉN A, ICHIJO H AND MIYAZONO K. (1992). Molecular cloning and characterization of the human and porcine transforming growth factor-β type III receptors. *Biochem. Biophys. Res. Comm.*, **189**, 356–362.

MOSES HL. (1992). TGF-beta regulation of epithelial cell proliferation. *Mol. Reprod. Dev.*, **32**, 179–184.

MOUSTAKAS A, LIN HY, HENIS YI, PLAMONDON J, O'CONNOR-MCCOURT MD AND LODISH HF. (1993). The transforming growth factor β receptors types I, II and III form hetero-oligomeric complexes in the presence of ligand. *J. Biol. Chem.*, **268**, 22215–22218.

NØRGAARD P, DAMSTRUP L, RYGAARD K, SPANG-THOMSEN M AND POULSEN HS. (1994). Growth suppression by transforming growth factor-β1 of human small cell lung cancer cell lines is associated to expression of the type II receptor. *Br. J. Cancer*, **69**, 802–808.

NØRGAARD P, HOUGAARD S, SPANG-THOMSEN M AND POULSEN HS. (1995). Transforming growth factor β and cancer. *Cancer Treat. Rev.*, **21**, 367–403.

PARK K, KIM S-J, BANG Y-J, PARK J-G, KIM NK, ROBERTS AB AND SPORN, MB. (1994). Genetic changes in the transforming growth factor β (TGF-β) type II receptor gene in human gastric cancer cells: Correlation with sensitivity to growth inhibition by TGF-β. *Proc. Natl Acad. Sci. USA*, **91**, 8772–8776.

PETTENGILL OS, SORENSON GD, WURSTER-HILL D, CURPHEY TJ, NOLL WW, CATE CC AND MAURER LH. (1980). Isolation and growth characteristics of continous cell lines from small-cell carcinoma of the lung. *Cancer*, **45**, 906–918.

ROBERTS AB AND SPORN MB. (1990). The transforming growth factor-βs. In *Handbook of Experimental Pharmacology*. Peptide Growth Factors and Their Receptors, Sporn MB and Roberts AB. (eds.) pp. 419–472. Springer: Heidelberg.

SAMBROOK J, FRITSCH EF AND MANIATIS T. (1989). *Molecular Cloning: A Laboratory Manual.* Cold Spring Harbor Laboratory: Cold Spring Harbor, NY.

SORENSON GD, PETTENGILL OS, CATE CC AND DELPRETE SA. (1984). Biomarkers in small cell carcinoma of the lung. In *Lung Cancer*, Aisner J (ed.) pp. 203–240. Churchill Livingstone: New York.

TEN DIJKE P, YAMASHITA H, ICHIJO H, FRANZÉN P, LAIHO M, MIYAZONO K AND HELDIN C-H. (1994). Characterization of type I receptors for transforming growth factor-β and activin. *Science*, **264**, 101–104.

VAN OBBERGHEN-SCHILLING E, ROCHE NS, FLANDERS KC, SPORN MB AND ROBERTS AB. (1988). Transforming growth factor β1 positively regulates its own expression in normal and transformed cells. *J. Biol. Chem.*, **263**, 7741–7746.

WANG X-F, LIN HY, NG-EATON E, DOWNWARD J, LODISH HF AND WEINBERG RA. (1991). Expression cloning and characterization of the TGF-β type III receptor. *Cell*, **67**, 797–805.

WIN KM, CHARLOTTE F, MALLAT A, CHERQUI D, MARTIN N, MAVIER P, PREAUX A-M, DHUMEAUX D AND ROSENBAUM J. (1993). Mitogenic effect of transforming growth factor-β1 on human ito cells in culture: evidence for mediation by endogenous platelet-derived growth factor. *Hepatology*, **18**, 137–145.

WRANA JL, ATTISANO L, CÁRCAMO J, ZENTELLA A, DOODY J, LAIHO M, WANG X-F AND MASSAGUÉ J. (1992). TGFβ signals through a heteromeric protein kinase complex. *Cell*, **71**, 1003–1014.

WRANA JL, ATTISANO L, WIESER F, VENTURA F AND MASSAGUÉ J. (1994). Mechanism of activation of the TGF-β receptor. *Nature*, **370**, 341–347.

YAMASHITA H, TEN DIJKE P, FRANZÉN P, MIYAZONO K AND HELDIN C-H. (1994). Formation of hetero-oligomeric complexes of type I and type II receptors for transforming growth factor-β. *J. Biol. Chem.*, **269**, 20172–20178.